# Magnitude of Aflatoxigenic *Aspergillus* Species, Level of Aflatoxin B1, and Associated Factors in Stored Feed at Poultry Farms in Dire Dawa, Ethiopia

**DOI:** 10.1155/2021/6638083

**Published:** 2021-10-21

**Authors:** Ambachew Motbaynor, Dawit Kassaye, Migbaru Keffale, Pawlos Wasihun

**Affiliations:** Haramaya University, College of Veterinary Medicine, P.O.Box 138, Dire Dawa, Ethiopia

## Abstract

Aflatoxin, the secondary toxic metabolite of *Aspergillus* species, particularly aflatoxigenic *Aspergillus flavus and parasiticus*, has a detrimental effect on poultry health and production. There exists some information gap about the magnitudes of aflatoxigenic *Aspergillus* species and aflatoxin in poultry feeds in the study area. Thus, the study was conducted to estimate the magnitude and assess the related potential factors of aflatoxigenic *Aspergillus* species with evaluations of the level of aflatoxin B1 in stored poultry feed at selected farms in Dire Dawa, Ethiopia. A cross-sectional study design was carried out on 374 poultry feed samples recruited by using a stratified simple random sampling technique. A pretested structured questionnaire was used to assess the level of knowledge and prevention practices associated with aflatoxin in poultry feed. The isolation of aflatoxigenic *Aspergillus* species was made by *Aspergillus flavus parasiticus* media, and aflatoxin B1 was estimated by aflatoxin B1 enzyme-linked immune sorbent assay. Results showed that the magnitude of aflatoxigenic *Aspergillus* species was 72.5% (95% CI: 67.6–76.9). The odds at which the species isolated were higher (*p* < 0.05) in feeds stored more than two months (AOR = 2.69), the presence of rodents in the storing room (AOR = 2.67), feeds having high moisture content (AOR = 1.5), and feed ingredient types (AOR = 4.3) compared to their counter parts. Only 34.4 and 32.8 percent of the respondents have better knowledge and apply prevention practice about fungal contamination and aflatoxin production in poultry feed, respectively. The occurrence of aflatoxigenic *Aspergillus* species in poultry feed was associated with the presence of rodents in the feed storing room with long storing period and high moisture contents of the feed. The knowledge and prevention practices employed by farm managers and workers about fungal contamination and aflatoxin in poultry feed are found low.

## 1. Introduction

The poultry sector continues to grow and industrialize in many parts of the world, and it is one of the key livestock subsectors of Ethiopia. Nowadays, poultry production is becoming commercially oriented as poultry enterprise requires small space or land allocation as compared to larger livestock types and crop enterprises. These large populations of commercially produced poultry rely on manufactured poultry feeds. However, the ingredients used for the manufacture of poultry feed are usually prone to aflatoxin contamination due to the environmental and storage conditions [1].

*Aspergillus flavus* (*A. flavus*) and *Aspergillus parasiticus(A. parasiticus)* are the widespread fungi isolated from a wide range of animal feeds and human foods, known to produce a highly carcinogenic, hepatotoxic, mutagenic, and immunosuppressive toxins called aflatoxins [2]. Aflatoxins are naturally occurring secondary metabolites of *Aspergillus* species that lead to global animal and public health problems acquired through the ingestion of fungi contaminated crops, animal feeds, and feed ingredients and through the accidental consumption of crop and crop products by humans. Especially in the tropical and subtropical regions of the world, the problem is more pronounced associated with the warm temperatures and humidity of the environment that favors the growth of the fungi [3].

Cereals and plant protein sources such as maize, wheat, soya bean, rice, wheat bran, and sunflower meals that are used in poultry feeds are the main sources of fungal contamination and aflatoxin production during preharvesting, harvesting, transportation, and storage [4]. *Aspergillus* germinates and grows better on feeds and grains at moisture levels of 15% or above with 25 to 35^o^c temperatures, but for maximum aflatoxin production, a moisture level above 17.5% and temperature of 27 to 30^o^C is required. Aflatoxins reduce the nutritional quality of ingredients by utilizing the nutrients present in the ingredients for their metabolism and propagation [5].

Aflatoxin B1 (AFB1) is the most prevalent and the greatest toxigenic threat from the four major types of the aflatoxin molecules, namely, B1, B2, G1, and G2 [6]. In poultry, AFB1 is rapidly absorbed from the small intestine into the mesenteric venous blood. B1 is metabolized into M1 and B2 in the liver, and the nicotinamide adenine dinucleotide phosphate-linked enzyme system reduces B1 and B2 to cyclopentanol and aflatoxinol in chicken. However, in laying chickens, aflatoxinol is the major metabolite in muscles and blood and both AFB1and aflatoxinol accumulate in eggs [4]. AFB1 impairs all important production parameters in poultry including feed intake, feed conversion efficiency, weight gain, pigmentation, egg production, and male and female reproductive performance [7]. Hence, regular monitoring of AFB1 in poultry feeds is an essential prerequisite to prevent aflatoxin build-up in poultry feeds.

The effect of aflatoxin on poultry depends on the age, physiological status, health, nutrition status, and times of exposure. It is very difficult to get rid of or to reduce the contamination once the aflatoxin is produced because this toxin has a high physical and chemical stability. Poultry feed contains 60–80% grains mainly maize, rice, and wheat and their by-products. In developing countries such as Ethiopia, the best-quality grains are used for human consumption and grains of poor quality are used as animal feeds which increases the contaminations of the feeds by the toxin. There is an information gap about the magnitudes of aflatoxigenic *Aspergillus* species and the levels of AFB1 in poultry feeds in Ethiopia, but there are reports on grains such as maize, wheat, and sorghum [8] and in dairy feeds in and around Addis Ababa [9] which show high prevalence of the fungus and the toxin in the grains and feed ingredients. Hence, this research work aims to estimate the magnitude and assess the related potential factors of aflatoxigenic *Aspergillus* species with evaluations of the level of AFB1 in stored poultry feed at selected farms in Dire Dawa, Ethiopia, from November 2019 to January 2020. The findings of this study are expected to assist poultry enterprises, farmers, investors, associations, and unions by revealing the magnitudes of aflatoxigenic *Aspergillus* species and aflatoxins in poultry feed and recommending optimum management options to reduce the contaminations of aflatoxigenic *Aspergillus* species and aflatoxin production in the poultry feeds. In this study, the term feed ingredient is used to indicate any cereal and grains such as maize, wheat bran, soya bean, nug cake, and bone and blood meal used to formulate a concentrate poultry feed. Those concentrate poultry feeds that are formulated (mixed) by the farm themselves were considered as home-formulated (home mixed) feeds, whereas those formulated by the factory as bulk and distributed to the users were termed as commercially formulated feeds. From the separate assessment questions set for knowledge and preventive practice on mould contamination and aflatoxin, those individuals who scored 50% and above of the knowledge questions and the preventive practices were considered knowledgeable and applied preventive practices, respectively.

## 2. Materials and Methods

### 2.1. Study Area

**Figure 1:**
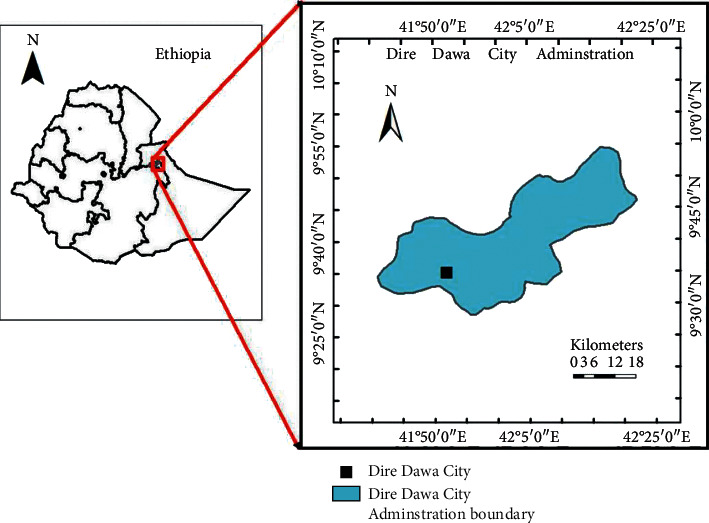
Dire Dawa city administration map.

The study was conducted at selected poultry farms in Dire Dawa city administration which is located 519 kilometers away from Addis Ababa. It is geographically located at 9°18′40″N 42°07′26″E and 950–1708 meter above sea level (Figure 1). The minimum and maximum annual temperatures of the city were 27 and 32^o^C, respectively, with an average humidity of 49.6%. The mean annual rainfall is about 637–734 mm [10]. There are 34 poultry farms in the study area; among these, 2 of them are broiler farms and 32 of them are layer farms which have 200–9000 heads of hens per farm.

### 2.2. Source of Feed

The source of feed was all stored poultry feed from selected poultry farms at Dire Dawa city administration.

### 2.3. Study Design

A cross-sectional study was conducted to evaluate the magnitude and associated risk factors for the occurrence of aflatoxigenic *Aspergillus* species and to determine the level of AFB1 among feeds in the study area.

### 2.4. Sample Size Determination

To evaluate the magnitudes of *Aspergillus* species in the poultry feed (first objective), the sample size was determined by a single proportion formula using an online software, Open Epi Version 3 (http://www.openepi.com), taking the assumption of 95% level of confidence, 5% absolute precision, and the respective expected prevalence indicated in Table 1, and the largest sample size 374 was set for the first objective.

To assess the associated risk factors for the occurrence of *Aspergillus* species in poultry feed (second objective), the sample size was determined by a double proportion formula using an online software, Open Epi Version 3 (http://www.openepi.com), taking the assumption of 80% power, 95% level of confidence, and the expected proportion of aflatoxigenic *Aspergillus* species in different potential sources indicated in Table 2, and the largest sample size is 132.

Therefore, to run the study simultaneously, the largest sample size from the two objectives was set, so 374 samples were required to undertake the study.

### 2.5. Sampling Techniques and Procedures

**Figure 2:**
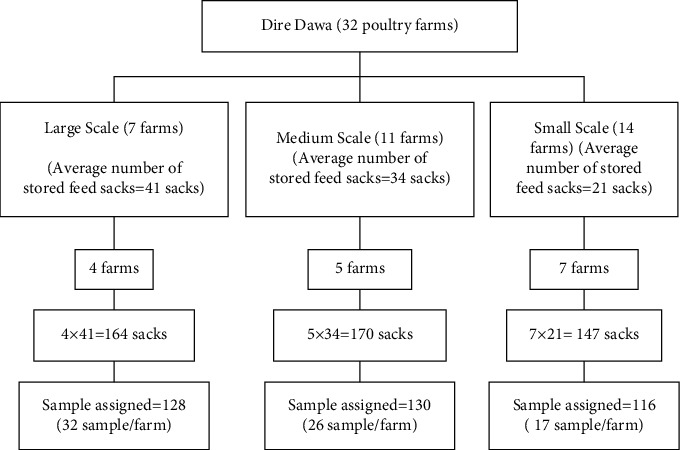
Schematic representation of the sampling procedure.

Comprehensive lists of poultry farms were obtained at the Dire Dawa city small-scale enterprise office to have the study frame. There were 32 poultry farms in the study area, and these farms were stratified based on the number of poultry flocks in to large-scale (≥1000 heads of birds), medium-scale (500–999 heads of birds), and small-scale (<500 heads of birds) farms to select participant farms. Sixteen (16) participant farms were selected randomly, and then, the sample size was allotted proportionally based on the number of sacks stored in each farm for each stratum (Figure 2). Then, the sample was taken by the systematic random sampling technique.

A semistructured questionnaire was designed and then administered to farm managers on the spot in the study farms. The questionnaire was designed to provide information on the knowledge and prevention practice of fungal contamination and aflatoxin production in the poultry feed at the farm level.

### 2.6. Sample Collection

**Figure 3:**
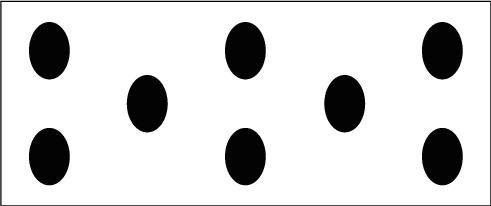
Sampling technique of feed on the lot.

100 g sample per 100 kg of feed was collected at eight different portions of the lot (sacks) by using a sterile metal probe (Figure 3). Then, they were homogenized together, and 20 g of feed was taken from this homogenized feed by using a sterile spoon and divided into three parts: part one for moisture content determination, part two for isolations of aflatoxigenic *Aspergillus* species, and part three for AFB1 analysis. Part one samples were analyzed immediately to avoid change in moisture content, while parts two and three samples were stored in a refrigerator at 4 °C using a polythene bag until the day of analysis, which was never more than seven days after collection [16].

### 2.7. Moisture Content of the Feed

Determination of moisture feed content was performed within the same days of sample collection, by the oven method. Two grams of feed in a dish was placed in an oven at 105°C overnight (16 hours). The feed was then weighed, and percentage moisture was calculated as tie percentage weight loss as follows:

(1) $$\begin{array}{c} \%\text{ moisture}=\frac{M_{o}-M_{1}}{M_{o}}\times 100, \end{array}$$

where *M*_*o*_ = initial weight in grams; *M*_l_ = final weight after drying in grams. Duplicate results for every sample were determined, and an average value was calculated per sample. The moisture contents of the feeds were categorized into three: low moisture content (<10%), medium moisture content (10–14%), and high moisture content (>14%) [17].

### 2.8. Isolation and Identification of the Aflatoxigenic *Aspergillus* Species

#### 2.8.1. Identification on Culture Media

Aflatoxin-producing *Aspergillus* species was isolated by *Aspergillus flavus parasiticus* agar media and prepared according to the manufacturer's instructions. One gram of the feed sample was aseptically transferred to a sterile test tube, and 9 milliliters of sterile distilled water was added to the feed and thoroughly mixed to perform a ten-fold serial dilution of the sample. Some 0.1 ml of the dilution was cultured by a spread-plate technique using a sterile bent glass rod and then incubated at a temperature of 27°C for 72 hours. The aflatoxin-producing *Aspergillus* species developed intense yellow orange color at the base of the colonies which is a differential characteristic for these species. Then, the isolates were enumerated and subcultured on Sabouraud dextrose agar media at 27°C in the dark for 5 days to obtain pure cultures. Suspicious colonies of *A. flavus* were identified by their greenish-yellow appearance and powdery texture with the reverse side golden to red brown or pale to yellow, while *A. parasiticus* were identified by their blue-green appearance with the reverse side having white to yellow appearance [18].

#### 2.8.2. Microscopic Identification

The microscopic features of the isolates were studied using the lactophenol cotton blue staining technique [19]. A drop of the stain was placed on a clean slide. A small part of the fungal cultures was removed and placed in the drop of the dye using a mounting needle. Then, the mycelium was spread by the same needle. Then, a cover slip was gently placed on the spread mixture with gentle digital pressure to eliminate air bubbles. Then, the slide was mounted and observed under an X40 objective lens. Identification of *A. flavus* was based on the presence of septate hyphae and rough and colorless conidiophores which end in a vesicle having the entire surface covered with either uni- or biseriate sterigmata (Phialid) and have bisereat metule, whereas *A. parasiticus* have unisereat metule and the vesicles are not covered entirely by uni- or biseriate sterigmata (Phialid) unlike *A.flavus*. Fungal taxonomic descriptions, identification keys, and Atlas were used as references [20].

#### 2.8.3. Determination of Aflatoxigenic *Aspergillus* Species

In order to determine toxigenic *Aspergillus* species, the ammonia vapor test method was employed as described in [21]. The organism was centrally inoculated on potato dextrose agar (PDA) plates. The plates were incubated in the dark at a temperature of 27°C for 5 days. After 5 days of incubation, a set of plates were inverted over 2 ml ammonium hydroxide for 15 minutes. Distilled water was used as a control instead of ammonium hydroxide. A change in color was used as a parameter to determine the toxigenic potentials of the *Aspergillus* species. Those strains having reverse turned pink color are recorded as positive (toxins producing strains). However, those without color change were recorded as negative (nontoxigenic strains).

### 2.9. Determinations of Aflatoxin B1 in Feed and Feed Ingredients by the Competitive ELISA Technique

#### 2.9.1. Sample Preparation (Extraction) and Assay Procedure

Crushed and homogeneous solid samples were formed as a fine-to-medium-fine powder by using a crusher; then, the required amounts of the crushed sample were taken depending on the sample; the homogeneous sample was weighed in a flask and mixed with the required amounts of 80% methanol based on the sample. The suspension was shaken intensively for 3 minutes to extract the aflatoxin. The suspension was then filtered via Watman paper No 1 after settling the solids for quantitative analysis. The filtrate (sample extract) was diluted in a new container with a different ratio with the sample diluents depending on the samples [22].

All the reagents were brought to room temperature, and the PBS-Tween packet was reconstituted. We placed one mixing well in a microwell holder for each standard and the sample to be tested and placed an equal number of antibody-coated microtiter wells in another microwell holder and then dispensed the sample diluents into each mixing well; after that, we added each standard and the prepared sample to the appropriate mixing well containing diluents. We transfered contents from each mixing well to a corresponding antibody-coated microtiter well and incubated at room temperature; then, we added the substrate solution and incubated again; after that, we added the stop solution and finally read the optical density of each microwell [22].

### 2.10. Statistical Analysis

The data were entered in to Microsoft® Excel sheet for Windows 2010. Then, they were exported into STATA statistical software version 14.0 (STATA corp., College Station, TX, USA) for data processing and analysis. Binary logistic regression was used to measure the strength of the association between the response variable with each predictor variable, and odds ratio was determined. Multivariable logistic regression analysis was run to assess the strength of association between all predictor variables with the response variable. Variables with *p* < 0.2 were selected as candidate to be included in the multivariate logistic regression analysis in an attempt to control for potential confounding variables, and adjusted odds ratio was determined. Colinearity between variables was also checked by standard error, and model fitness was assured by the Hosmor and Lemshow test. The AFB1 concentrations of the positive feed samples were calculated by using Graph Pad Prism 7 software. Throughout the data presentation, *P* value less than 0.05 (i.e., *p* < 0.05) was considered statistically significant.

## 3. Results

### 3.1. Magnitudes of Aflatoxigenic *Aspergill*us Species in Poultry Feed

Out of 374 poultry feed samples cultured, 72.5% (95% CI, 67.6–76.9) were positive for aflatoxigenic *Aspergillus* species. From these, 48.9% (95% CI, 43.8–54.1) were *A. flavus* and the remaining 23.6% (95% CI, 19.3–28.2) were contaminated with *A. parasiticus*. Among those samples with either of the species, 69.4% (95% CI, 63.5–74.8) were toxigenic (Table 3).

From the feed types analyzed, high magnitudes of aflatoxigenic *Aspergillus* species contamination were shown in the mixed feeds (81.7%) whereas low magnitudes were seen in wheat bran (35.6%). High fungal contamination (81.6%) was observed among feeds stored for more than 2 months, but low fungal contamination (58.1%) was seen in feeds stored for less than a month. More fungal contamination (92.7%) was seen in feeds having high moisture content (>14%), while low fungal contamination (35%) was seen in feeds with low moisture content (<10%) (Table 4).

### 3.2. Factors Independently Associated with Aflatoxigenic *Aspergillus* Species Contamination in Poultry Feed

Aflatoxigenic *Aspergillus* species contamination was found independently associated with the feed storing period, source of feed, presence of rodents in the feed storing room, moisture contents of the feed, and feed ingredient types (*p* < 0.05) (Table 5).

More specifically, the likelihood of aflatoxigenic *Aspergillus* species contamination in feeds stored one up to two months was nearly two times (AOR 1.99, 95% CI: 1.05–3.76) that of those feeds stored less than one month. Feeds which were stored more than two months were 2.69 times (AOR 2.69, 95% CI: 1.08–6.69) more likely to encounter aflatoxigenic *Aspergillus* species contamination than feeds that were stored less than one month. Feeds which were stored in rooms having rodents were 2.67 times (AOR 2.67, 95% CI: 1.3–5.39) more likely to encounter aflatoxigenic *Aspergillus* species contamination than those which were stored in rodent-free rooms. Home-mixed (farm) feeds were 27% (AOR 0.73, 95% CI: 0.39–1.1) less likely exposed to aflatoxigenic *Aspergillus* species contamination than commercially formulated feeds. The odds of fungal contamination in feed ingredients such as maize (AOR 4.3, 95% CI: 1.6–11.5), soya bean (AOR 3.49, 95% CI: 0.6–5.4), and nug seed cake (AOR 3.45, 95% CI: 0.6–5.3) were more than three times those in wheat bran. Feeds which had a moisture content of less than 10% (low moisture content) were 81% (AOR 0.19, 95% CI:0.1–0.25) less likely exposed to aflatoxigenic *Aspergillus* species contamination than feeds which had medium moisture content (10–14%), whereas feeds which had high moisture content (>14%) were 1.5 times (AOR 1.5, 95% CI: 0.9–4.6) more likely to be exposed to aflatoxigenic *Aspergillus* contamination than those which had medium moisture content (10–14%). However, aerations or ventilation was not found to be an independently associated factor for aflatoxigenic *Aspergillus* species contamination in feed (*p* < 0.05) (Table 5).

### 3.3. Mean Aflatoxin B1 Levels of Mixed and Individual Ingredients of Poultry Feeds

**Figure 4:**
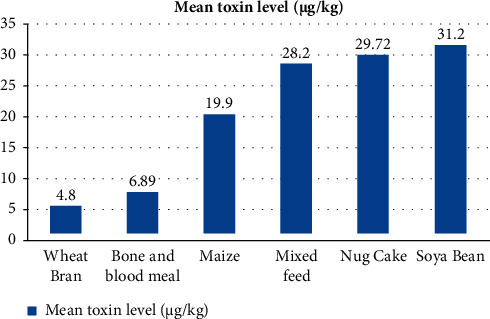
Mean AFB1 levels of stored poultry feed and feed ingredients in farms at Dire Dawa, Ethiopia.

From all mixed and individual ingredients of poultry feeds analyzed for AFB1, minimum and maximum mean AFB1 levels were 4.8 *μ*g/kg and 31.2 *μ*g/kg for wheat bran and soya bean, respectively (Figure 4).

### 3.4. Mean AFB1 Levels of Poultry Feeds and Daily Toxin Intake of Hens with Respect to Feed Source

Based on the ration formulation that the farms used to prepare their poultry feeds described below, the minimum and maximum AFB1 level per kg of feed was 7.34 *μ*g/kg and 41.0 *μ*g/kg from home-mixed feeds and commercially formulated feeds, respectively, and the minimum and maximum AFB1 intake per hen per day were 0.92 *μ*g and 4.92 *μ*g, respectively (Table 6).

(2) $$\begin{array}{c} \text{Ration formulation per }100\text{ kg of feed }=\;45\text{\% maize }+\;16\text{\% nug cake }+\;10\text{\% soya bean }+\;20\text{\% bone and blood meal }+\;7\text{.}5\text{ \% wheat bran }+\text{ 0.}5\text{\% salt}+1\text{\% vitamin.}\\ \text{Total toxin intake/hen/day}=\left(\text{daily feed intake/hen}\right)*\left(\text{total toxin in }\mu \text{g/}100\text{ kg of feed}\right)\text{.} \end{array}$$

Among home-mixed feeds, 75.0% were within the FDA AFB1 standard permissible limits (≤20 *μ*g/kg) and only 25.0% exceeded the maximum FDA AFB1 permissible limits standards (>20 *μ*g/kg), whereas in commercially formulated feeds, 62.5% of feeds fulfilled FDA permissible limits, and some 37.5% of the commercial feeds exceeded the maximum FDA permissible limits.

### 3.5. Knowledge Assessments of Farm Managers, Professionals, and Daily Poultry Farm Workers on Fungal Growth and Aflatoxin in Poultry Feeds

Sociodemographic characteristics among the farm managers, professionals, and daily farm workers were observed. From all the respondents, 62.5% were 20–30 years old and 37.5% were females. Academically, 39.1% of the respondents were technical, vocational, and education training college (TVET) graduates and 28.1% were university graduates. From all the respondents, only 34.4% and 20.3% had knowledge about fungal growth and aflatoxin production in poultry feeds, respectively. Regarding the prevention practice, only 32.8% applied prevention practice on fungal contamination and aflatoxin production in poultry feeds (Table 7).

## 4. Discussion

The study showed that the magnitude of aflatoxigenic *Aspergillus* species contamination in poultry feed from poultry farms in Dire Dawa administrative council was 72.5%. The finding is in agreement with 77.9% in Nigeria [23], 73.1% in Pakistan [24], 65% in Tanzania [25], 64.29% in Sudan [26], and 57% in Myanmar [27], but it is lower than 83% in Cameroon [12]. However, it is higher than 46% in France [28], 35% in Malawi [29], and 30% in South Africa [30].

Low level of fungal contamination in this study as compared to Cameroon may be due to the environmental factors and the feed ingredient type used. Cameroon, where the studies were conducted, had an average temperature range of 22–30 0c and 60–80% humidity [12], which is a conducive environmental condition for fungal growth and aflatoxin production. In addition, maize and peanut are the main constituents of poultry feed in Cameroon [12] which are the most preferable substrates for fungal growth and aflatoxin production due to high contents of soluble sugars in maize and high lipid content in peanut [31], [32].

The high occurrence of fungal contamination in this study compared with South Africa and France may be due to the presence of well-developed commercial farming systems in France and South Africa [28, 33]), which reduces the fungal contamination and aflatoxin production by reducing the factors that promote the contamination of fungus and toxin production by pre- and postharvest crop management practices such as using crop hybrids that are less susceptible to fungal contamination and aflatoxin production, reducing physical damage to crops at harvest, pest management, good sanitation, and improved feed storing practices [34].

From the isolated *Aspergillus* species, 69.4% was aflatoxigenic. The result is comparable with 64.29% in Sudan [26] and 60% in Pakistan [4], but it is lower than 98% from Tamil Nadu, India [35], 88% from cereals in Ethiopia [8], and higher than 42% in Zaria, Nigeria [11], 35% in Malawi [29], and 16.1% in Ilaro, Nigeria [36]. These toxigenic variations may be due to variations in factors that determine the productions of aflatoxin in the respective countries such as temperature, humidity, storing periods, and moisture contents of feed together with the feed type they used.

From the feed ingredients, the maximum mean AFB1 was recorded from soya bean while the minimum mean AFB1 level was registered in wheat bran. This finding is comparable with that in Ethiopia [9, 37]. The maximum mean AFB1 in soya bean may be due to its high lipid content which significantly stimulates fungal growth and AFB1 production [31], and the minimum mean AFB1 in wheat bran may be due to its low moisture content and poor nutritional composition as compared to other feed ingredient types [15].

In this study, concentrated (mixed) feeds were more exposed (81.7%) to aflatoxigenic *Aspergillus* species contamination than feed ingredients. In feed ingredients, aflatoxigenic *Aspergillus* species contamination was seen as 78.6% in maize, 74.4% in soya bean, 73.9% in nug cake, 60% in bone and blood meal, and 35.6% in wheat bran as their exposing order. This finding is comparable with 80.9% in mixed feed, 65.3% in maize, 48.6% in soya bean, and 18.5% in wheat bran in India [38] but lower than 100% in mixed (concentrated) feed, 100% in maize, and 86.67% in soya bean at Bangladesh [39]. High fungal contamination in concentrated feed might be due to fact that concentrated feeds have high fat, carbohydrate, and protein content which is suitable for fungal growth and aflatoxin production [40]. From individual feed ingredients aflatoxigenic *Aspergillus* species contamination is high in maize and low in wheat bran. High fungal contamination in maize might be due to the presence of high soluble sugars (glucose, sucrose, and maltose) in maize which are excellent substrates for fungal growth [32]. Low fungal contamination and toxin production in wheat bran might be due to the fact that most wheat bran available as an animal feed is a by-product of wheat milling in the preparations of flour used for human consumption. In doing so, sorting and cleaning is carried out. Sorting and cleaning of grains to remove damaged ones is a process which has been shown to significantly reduce the aflatoxin level [32].

This finding showed that 25.0% of home-mixed feeds and 37.5% of commercially formulated feed exceeded the maximum FDA AFB1 permissible standard limits (>20 *μ*g/kg). The results are consistent with other studies conducted elsewhere [41, 42]. That more commercially formulated feeds exceeded FDA permissible limits than home-mixed feeds may be attributed to the fact that, in commercially formulated feeds, they are mixed during buying, thus creating favorable conditions for fungal contamination and aflatoxin production during transportation and storage, whereas in home-mixed feeds, the farms buy feed ingredients rather than mixed feeds and mix by themselves on farm; as a result, feeds are stored in the form of feed ingredients rather than mixed feeds and there may be cleaning and sorting feed ingredients during mixing. Therefore, preprocessing of ingredients helps to reduce fungal contamination and aflatoxin production in home-mixed feeds [43].

Based on this study, the minimum and maximum AFB1 intake per hen per day was 0.92 *μ*g and 4.92 *μ*g, respectively, which was low. However, low doses of continues consumptions of AFB1 by the hen may have far-reaching effects on its health and overall performance. Since chronic exposure to small amounts of AFB1causes morphological damage to small intestinal mucosal linings, the absorptive surface of the small intestine will decrease exposing the hens to feed deficiency, increasing disease susceptibility and vaccine failure [15].

Among the aflatoxigenic *Aspergillus* species contamination-related factors considered in this study, the feed storing period, source of feed, presence of rodents in the feed storing room, moisture contents of the feed, and feed ingredient types were found to be independently associated with the contamination of aflatoxigenic *Aspergillus* species. Hence, according to the current finding, feeds stored in rodent-infested rooms were 2.67 times more likely to encounter aflatoxigenic *Aspergillus species* contamination than those stored in rodent-free rooms. The observed contamination may be attributed to the presence of rodents or insects in the feed (feed storing room) which predispose the feeds to fungal contamination and aflatoxin production by transport primary inoculums as a vector, disseminate spores within the feed, and facilitate colonization and infection by injuring the feedstuffs and increasing the moisture contents of the feed by their by-products [44]. Home-mixed feeds were 27% less likely exposed to aflatoxigenic *Aspergillus* species contamination than commercially formulated feeds bought from feed vendors. The findings are in line with the reports of Aliyu in Nigeria [45]. This might be due to that concentrated feeds were more prone to fungal contamination and aflatoxin production during transportation and storing time than feed ingredients because concentrated feeds are crushed and nutrient rich, which creates a suitable condition for fungal contamination and toxin production than feed ingredients. The justification is further supported by the recommendation from the USAID [46] that 23% aflatoxin production can be reduced by home preparation of feed.

The likelihood of aflatoxigenic *Aspergillus* species contamination in feeds stored for one to two months was nearly two times that in feeds stored for less than one month, whilst feeds which were stored for more than two months were 2.69 more likely to encounter aflatoxigenic *Aspergillus* species contamination than feeds that were stored for less than one month. The findings are comparable with the reports in [15] at Hawasa, Ethiopia. The increment of fungal contamination with increasing feed storing time might be due to the increment of the probability of the feed damaged by insects and rodents which increases the moisture contents of the feed that creates conducive environment for fungal contamination and aflatoxin production [47].

Feeds which have low moisture content (<10%) were 81% less likely to encounter aflatoxigenic *Aspergillus* species contamination than feeds which had medium moisture content (10–14%), and feeds which had high moisture content (>14%) were 1.5 times more likely to be exposed to aflatoxigenic *Aspergillus* species contamination than feeds which had medium moisture content (10–14%). The finding is consistent with other previous studies conducted elsewhere [12, 17].

The assessment of the awareness on fungal contamination and aflatoxin production in feeds revealed levels of 34.4 and 20.3 percent, respectively, among farm owners, factory managers, and workers. From all the respondents, only 32.8 percent applied prevention practice on fungus contamination. The result is higher than 10% knowledge and 30% prevention practice in Addis Ababa [15]. The observed better knowledge and prevention practice against fungal contamination and aflatoxin in feed compared to previous studies might be due to increased access to information on aflatoxin and its health impacts from different medias which create awareness about fungal contamination and effects of aflatoxin in feed and animal products presently than in the past.

## 5. Conclusions

The magnitudes of aflatoxigenic *Aspergillus* species in poultry feed were found high, and *A. flavus* is the more frequently isolated aflatoxigenic *Aspergillus* species than *A. parasiticus.* Fungal contamination in poultry feed was associated with the presence of rodents in the feed storing room, longer storing period, and high moisture contents of the feed. The highest rate of fungal contamination was observed in maize followed by soya bean and nug cake due to high soluble sugar in maize and high lipid content in soya bean and nug cake, whereas the lowest rate was found in wheat bran associated with its low moisture content and poor nutritional composition as compared with others. As a result of cleaning, sorting, and shorter storing period of feed, lower rate of aflatoxigenic *Aspergillus* species contamination was observed in home-mixed (farm) feeds than commercially formulated ones bought from feed vendors. A maximum mean aflatoxin level was measured in soya bean, and a minimum mean aflatoxin level was measured in wheat bran. Nearly one-third of the feeds showed the aflatoxin level beyond the maximum FDA permissible level. Generally, biological, environmental, and feed-related factors were found to be key determinant factors associated with the contamination of aflatoxigenic *Aspergillus* species in poultry feeds. The knowledge levels and adapted prevention practices about fungal contamination and aflatoxin production among poultry industry stakeholders were very low.

In line with the abovementioned conclusion, the following recommendations are forwarded:

- Awareness creation about the impact of aflatoxin in poultry health and production, together with prevention methods, should be extended to all stakeholders along the poultry value chain
- The farm owners should be encouraged to prepare their poultry feeds by themselves rather than buying commercially prepared concentrated feeds in bulk from feed vendors
- The farm owners should dry the feeds and feed ingredients well before storage and avoid long periods of feed storage
- Feed storing room should be free of insects and rodents, well ventilated, and cleaned before restocking the feed, and the first in first out principle of the feed utilization should be implemented
- Surveillance and scheduled aflatoxin monitoring programs should be implemented in poultry farms and commercial poultry feed processing factories
- There have to be country-based set standards on maximum aflatoxin permissible limit in poultry feeds
- Extension programmes targeting farm owners, managers, and workers about knowledge of fungal contamination and aflatoxin in feed need be enhanced

## Figures and Tables

**Table 1:** Required sample size for determining magnitudes of aflatoxigenic *Aspergillus* species.

Study area	Magnitude (%)	Calculated sample size	Reference
Zaria, Nigeria	42	**374**	[11]
Cameroon	83	217	[12]
Kenya	78	264	[13]

Bold indicates the larger sample size from the three calculated sample sizes of the respective study area.

**Table 2:** Required sample size for different factors.

Factors	Proportions (%) in different groups	Calculated sample size	Reference
Storage time	Up to 30 days (29.7%)	66	[14]
	31–90 days (66.6%)		
Feed ingredient 1	Noug cake (58.3%)	78	[15]
	Wheat bran (25%)		
Feed ingredient 2	Wheat bran (4.4%)	132	[14]
	Concentrate feed (22.5%)		

**Table 3:** Magnitudes of aflatoxigenic *Aspergillus* species and frequency distributions of *A. flavus* and *A. parasiticus* in poultry feed at Dire Dawa, from November 2019 to January 2020.

*Aspergillus* species	Sample examined, *n* = 374		Toxigenic		Toxigenic potential (%) (95% CI)
	Sample positive	Magnitude (%) (95% CI)	Yes	No	
*A. flavus*	183	48.9 (43.8–54.1)	134	49	73.2(66.2–79.5)
*A. parasiticus*	88	23.6(19.3–28.2)	54	34	61.4 (50.4–71.6)
Total	271	72.5 (67.6–76.9)	188	83	69.4 (63.5–74.8)

**Table 4:** Magnitudes of aflatoxigenic *Aspergillus* species among the study variables in poultry feed at Dire Dawa, from November 2019 to January 2020.

Variables	Category	Aflatoxigenic *Aspergillus* species		Total	Magnitude (%) (95% CI)
		Yes	No		
Feed storage period	<1 month	61	44	105	58.1 (48.1–67.7)
	1-2 month	108	36	144	75.0 (67.1–81.8)
	>2 month	102	23	125	81.6 (73.7–88.0)
Feed source	Home mixed	157	66	223	70.4 (64.0–76.3)
	Commercially formulated	114	37	151	75.5 (67.8–82.1)
Room aeration	Yes	81	46	127	63.8 (54.8–72.1)
	No	190	57	247	76.9 (71.2–82.0)
Presence of rodent	Yes	220	57	277	79.4 (74.2–84.0)
In the store	No	51	46	97	52.6 (42.2–62.8)
Feed ingredients	Maize	66	18	84	78.6 (68.3–86.8)
	Bone and blood meal	18	12	30	60 (40.6–77.3)
	Soya bean	29	10	39	74.4 (57.9–87.0)
	Wheat bran	16	29	45	35.6 (21.9–51.2)
	Nug cake	17	6	23	73.9 (51.6–89.8)
	Mixed feed	125	28	153	81.7 (74.6–87.5)
Moisture content	Low (<10%)	36	67	103	35 (25.8–45.0)
	Medium (10–14%)	146	29	175	83.4(77.1–88.6)
	High (>14%)	89	7	96	92.7 (85.6–97.0)

**Table 5:** Multivariate analysis results of associated factors for aflatoxigenic *Aspergillus* species contamination in stored poultry feed at farms in Dire Dawa, Ethiopia.

Variables	Category	Fungal contamination		Total	AOR (95% CI)	*p* value
		Yes	No			
Storing	<1 month	61	44	105	1.00	
	2 month	108	36	144	1.99 (1.05–3.76)	0.034
	>2 month	102	23	125	2.69 (1.08–6.69)	0.033
Moisture	Low (<10%)	36	67	103	0.19 (0.06–0.25)	<0.001
Content	Medium (10–14%)	146	29	175	1.00	
	High (>14%)	89	7	96	1.5 (1.1–4.6)	0.042
Source of	Home mixed	157	66	223	0.73 (0.39–0.99)	0.001
Feed	Commercially	114	37	151	1.00	
Aeration	No aeration	81	46	127	1.7 (0.9–3.26)	0.1
	There is aeration	190	57	247	1.00	
Rodent	There is rodent	220	57	277	2.67 (1.3–5.39)	0.006
	No rodent	51	46	97	1.00	
Feed	Maize	66	18	84	4.3 (1.6–11.5)	0.003
Type	Bone and blood meal	18	12	30	1.87 (0.89–3.5)	0.25
	Soya bean	29	10	39	3.49 (1.6–5.4)	0.031
	Wheat bran	16	29	45	1.00	
	Mixed feed	125	28	153	6.3 (3.25–16.2)	<0.001
	Nug cake	17	6	23	3.45 (0.6–5.3)	0.008

**Table 6:** Mean AFB1 level of home-mixed and commercially formulated poultry feeds.

	List of farms	Mean toxin levels of each feed ingredients per farm (*μ*g/kg)					Mean AFB1 of feed	Daily feed intake per hen (gm)	Daily toxin intake per hen (*μ*g)
		Maize	Bone meal	Soya bean	Wheat bran	Nug CaK			
Mixed feeds by mixing	Farm 1	19.05	0	28.93	0	38.08	17.21	120	2.12
	Farm 2	28.07	11.8	38.3	5.53	10.0	18.74	115	2.39
	Farm 3	32.31	8.3	53.2	7.5	77.1	35.68	120	4.13
	Farm 4	17.5	4.75	29.2	9.00	0	7.34	125	0.92
	Farm 5	19.5	19.1	4.9	0	34.0	15.5	120	2.19
	Farm 6	12.82	6.4	45.97	0.6	0	13.2	125	1.55
	Farm 7	11.9	2.67	10.25	4.75	47.5	15.4	125	1.86
	Farm 8	18.05	2.14	27.63	2.1	31.08	19.7	125	2.05
Used commercially feed from feed	Farm 9						41.0	120	4.92
	Farm 10						34.7	120	3.72
	Farm 11						17.2	120	3.38
	Farm 12						32.4	130	4.21
	Farm 13						19.8	125	2.47
Formulated vendors	Farm 14						18	130	2.34
	Farm 15						18.4	125	3.55
	Farm 16						19.1	120	3.13

**Table 7:** Frequency distributions of sociodemographic, knowledge, and prevention practice assessments on fungal contamination and aflatoxin production in poultry feed.

Variable	Category	Total	Percent (%)
Sex	Male	40	62.5
	Female	24	37.5
Age (year)	20–30	40	62.5
	Above 30	24	37.5
Education	High-school certified	21	32.8
	TVET graduate	25	39.1
	University graduate	18	28.1
Occupation	Farm manager	16	25
	Professionals	35	54.7
	Daily farm worker	13	20.3
Knowledge about fungal contamination	Yes	22	34.4
	No	42	65.6
Knowledge about aflatoxin	Yes	13	20.3
	No	51	79.7
Fungal contamination prevention practice	Yes	21	32.8
	No	43	67.2

## Data Availability

The raw data used to support the findings of this study are available from the corresponding author upon request.
